# Bibliographical Notices

**Published:** 1846-12

**Authors:** 


### BIBLIOGRAPHICAL NOTICES.

*Chemistry of the four Seasons, Spring, Summer, Autumn and Winter; an essay principally concerning natural phenomena, admitting of interpretation by Chemical Science, and illustrating passages of Scripture, by* THOMAS GRIFFITHS, *Prof. of Chemistry in the Medical College of St. Bartholomew's Hospital, etc.* Philadelphia, Lea & Blanchard. 1846, 12mo. pp. 450.

It is gratifying to see evidences of a growing relish, among the intellectual classes of society, for scientific truth. We live in a world of wonderful phenomena and mysterious agencies: They need not be sought after, for they exist around us on every side—in the atmosphere we breathe, the plants we crush under our feet, the earth which we spurn for it grossness, in the animal creation from the Leviathan to the animalcule, and last, but most emphatically, in our own bodies, so "fearfully and wonderfully made." Here is abundant scope for the efforts of genius, and an ample range for the poetic imagination. Religious aspirations and devotional gratitude may find in the revelations of science, fruitful occasions for culture and gratification, exhibiting, as they do, constant illustrations of the power, wisdom, and beneficence of Deity. How rational and satisfactory appear the calm, ennobling pursuits of science, when contrasted with the frivolous extravagances of fiction, or the productions of an aimless sentimentality! The multiplication of books which teach mankind to regard the common phenomena of nature with the eye of philosophy, affords one of the best proofs, that notwithstanding the surface of the popular literature of the day is covered with worthless and pernicious publications, there is below the surface, an under current adapted to a pure and healthy tone of thought and sentiment.

The title page copied at the head of this article sufficiently expresses the objects of the work, and the subjects of which it treats. It is a token from the laboratory, designed for the parlor. The discourses are adapted to the comprehension of those not familiar with the technicalities and details of chemistry, and are written in a pleasing style. The mechanical execution of the work is neat and tasteful. Altogether it is a beautiful volume, well adapted for a Christmas offering, which shall convey a delicate compliment to the intellectual taste of the recipient.

---

*The Writings of Hippocrates and Galen, epitomised from the original Latin translations, by* John Redman Coxe, M. D. *etc. Multa Renascentur.* Philadelphia, Lindsay & Blackeston, 1846, 8vo. pp. 681.

This volume promised some time since by the venerable Dr. Coxe, is now offered to the medical public; and as we think, will be received by the profession as a most acceptable offering. As remarked by the translator, the names of these fathers of medicine are household words. We speak of them, refer to them, quote them, without ever having perused their writings. It is indeed strange that full translations have never been made for the English reader. It is not a motive of mere useless curiosity which should prompt us to make ourselves familiar with primeval medical literature. It may teach us some valuable lessons. We shall probably be led to place a juster estimate on our present knowledge, by comparison with the observations and reflections of ancient authors; and thereby learn to contemplate the present condition of medicine with proper humility. Great as has been the progress of the various branches of Medical Science, and rapid as are its onward strides at the present moment, it is neither wise nor prudent to forget that the sagacity of the earliest practitioners penetrated, in many respects, the mysterious processes of life, and the operations of remedies, nearly as far as ages of industrious exertions have been able to carry us. We are too prone to plume ourselves upon what the intellect by long continued and successive efforts has been able to accomplish, without taking due cognizance of the limitations of human knowledge. These limitations in medicine are practically of no little importance to be considered. It is nearly as essential to the practitioner to appreciate what is *not* known as to know what *is* known. Will it not, therefore, be an useful discipline to go back occasionally to the early fathers, and study medicine as it was in the early periods of its history!

Dr. Coxe has performed his task *con amore*. It has been with him a labor of love. The undertaking manifestly was assumed from a profound veneration of the writings which he has epitomised. With such a spirit, and with his experience and learning, the duty could not but be well performed.

We consider the book a valuable addition to a medical library, and we presume the profession generally will participate in this sentiment, and act accordingly.

---

***Minor Surgery**; or Hints on the every-day duties of the **Surgeon**.* By Henry H. Smith, M. D., *Lecturer on the **Practice of Surgery**, &c. Second Edition, with numerous additions; **Illustrated with** 227 engravings.* Philadelphia, Ed. Barrington & Geo. D. Haswell, 1846; pp. 377, 8vo.

The work is divided into five principal parts,—1. Dressings and bandages; 2. Mayor's handkerchief system; 3. Apparatus for fractures; 4. Apparatus for dislocations; 5. Minor surgical operations,—and embodies a large amount of practical information upon "every-day" surgery in a small compass.

The drift of the author seems to be not only to furnish a short vade mecum, but also to so simplify ordinary surgical instruments and practice as that a man may carry in his saddle bags a complete armamentaria chirurgica, from a thumb lancet to a fracture bed; in all of which he discovers his peculiar American character and renders his book to us the more acceptable. We love simplicity and brevity, and we commend every attempt to dispense with the complicated *appareil* in the treatment of surgical accidents. But simplicity certainly is not the *sole* object attained, nor indeed the *principal*—the great aim of the clever surgeon should be to make his apparatus *effective*, and that apparatus which accomplishes this end most perfectly is always best, however complex.

M. Mayor, a surgeon of Lausanne, has persuaded himself and a few others, including our author, that a *common handkerchief* by ingenious foldings and reversions, and various modes of application, may be made to retain properly all the forms of fracture in every part of the body. Sixty pages are devoted to this new and somewhat persecuted mode of bandaging, wherein are shown how the handkerchief can be made to tie up a broken head, arm, leg, etc. etc., in a manner, as we humbly think, most slovenly and insufficient. Thus we can scarcely believe that any man having had practical evidence of the difficulty of retaining a fractured clavicle in situ during the first three or four weeks necessary to its union, in children and adults, by night and by day, would ever think of trusting to handkerchiefs alone in the manner represented in the cut, when either Boyer's or Fox's apparatus could be had—and they may always be had for an hour's work by a common seamstress.

The same remarks apply equally to almost every other limb in the body—we have many better modes of dressing, and we sincerely hope, for the sake of the unfortunate patient, and for the sake of the surgeon's peace and reputation, that no one will be induced to adopt this very absurd "handkerchief system." It may do well enough—we think it will—among the crooked, vilely mis-shapen and drivelling Cretins, with whom M. Mayor has to do, along the valleys of the Jura and the Alps, for with them it matters little how the bones unite—they can scarcely be made more distorted than they were before; but it will never answer, Mr. Smith, in this land of comely forms and of law.

It is a most excellent book, notwithstanding this unfortunate blemish, and will, we have no doubt, soon supersede those transatlantic works of the same class, such as Castle, Bourgery, Cutler, Coster, &c., whose chief enchantment is derived from the distance of their authors. We recommend the work especially to those general practitioners of medicine and surgery, who, from their situation are compelled to make the minor, but do not intend to undertake the capital surgical operations. F. H. H.

